# Persistent trigeminal artery linked with partial duplication of the anterior communicating artery

**DOI:** 10.1007/s00276-025-03687-9

**Published:** 2025-07-16

**Authors:** George Triantafyllou, Panagiotis Papadopoulos-Manolarakis, George Tsakotos, Anastasia Triantafyllou, Maria Piagkou

**Affiliations:** 1 Department of Anatomy, Faculty of Health Sciences, School of Medicine, National and Kapodistrian University of Athens, 75 Mikras Asias str., Goudi, 11527 Athens, Greece; 2 Department of Neurosurgery, General Hospital of Nikaia-Piraeus, Athens, Greece

**Keywords:** Persistent trigeminal artery, Anterior communicating artery, Carotid-vertebrobasilar anastomosis, Anatomy, Variation

## Abstract

Variations of the cerebral arterial circle are frequently observed due to the progressively increasing utilization of diagnostic imaging techniques. Among these variations, the persistence of embryonic vessels, such as the persistent trigeminal artery (PTA), is rare yet holds significant clinical relevance. This presentation details a distinctive case of the PTA coexisting with a partial duplication of the anterior communicating artery (AComA), as identified through magnetic resonance angiography (MRA) in a 41-year-old female patient. The PTA was documented as a vessel linking the basilar artery to the cavernous segment of the left internal carotid artery. The AComA exhibited two branches emanating from the left anterior cerebral artery (LACA), which fused to form a single vessel that anastomosed with the right anterior cerebral artery (RACA). The coexistence of the PTA and AComA variant accentuates the imperative for clinicians to consider such rare anatomical configurations, mainly when performing procedures in proximity to the gasserian ganglion, cavernous sinus, or anterior circulation. The application of three-dimensional imaging remains vital for ensuring precise diagnosis and effective treatment planning.

## Introduction

Variations of the cerebral arterial circle are frequently noted due to the substantial volume of diagnostic imaging scans conducted in this region. Among the most intriguing variants, the persistence of embryonic vessels is seldom documented and possesses significant embryological implications. Several types of carotid-vertebrobasilar anastomosis are identified based on the fetal vessels, arranged from caudal to cranial: proatlantal intersegmental, hypoglossal, otic, and trigeminal arteries. The most described ones are the proatlantal intersegmental that represents an anastomosis between the external or internal carotid arteries and the V3 segment of the vertebral artery, while the hypoglossal arises from the cervical internal carotid artery and enters the posterior cranial fossa via the hypoglossal canal [8]. Typically, these arteries undergo regression or fusion with one another to establish the adult arterial system; nevertheless, any deviation from these intricate processes results in the persistence of these embryological structures [8]. The persistent trigeminal artery (PTA) is the most common persistent vessel, with an estimated pooled prevalence of 0.3% [2]. Nevertheless, the vessels of the cerebral arterial circle exhibit substantial morphological variability. For example, the anterior communicating artery (AComA) variants are associated with a pooled prevalence of 33% [7]. Herein, we describe the coexistence of the PTA and AComA variants in the same patient.

## Anatomic variation

In an angiographic study using an archived magnetic resonance angiography (MRA) set, the scan of a 41-year-old woman was analyzed. This set was obtained from the General Hospital of Nikaia-Piraeus following ethical approval from the appropriate authorities (protocol number: 56485, approved on: 13.11.2024). The scans were documented using Horos software version 3.3.6 (Horos Project). Evidence was gathered from the multiplanar reconstruction of the axial, coronal, and sagittal slices, as well as their three-dimensional volume reconstruction.

**Fig. 1:**
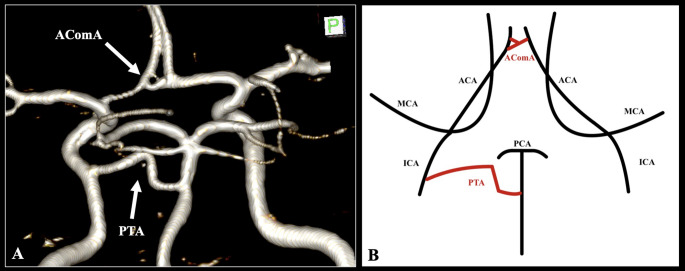
The cerebral arterial circle of the patient. Three-dimensional reconstruction (**A**) and schematic representation (**B**). The persistent trigeminal artery (PTA) is indicated as the anastomosis between the basilar artery (BA) and the internal carotid artery (ICA). This variant is associated with partially duplicating the anterior communicating artery (AComA) (**A**, **B**). ACA, anterior cerebral artery; MCA, middle cerebral artery; PCA, posterior cerebral artery

**Fig. 2:**
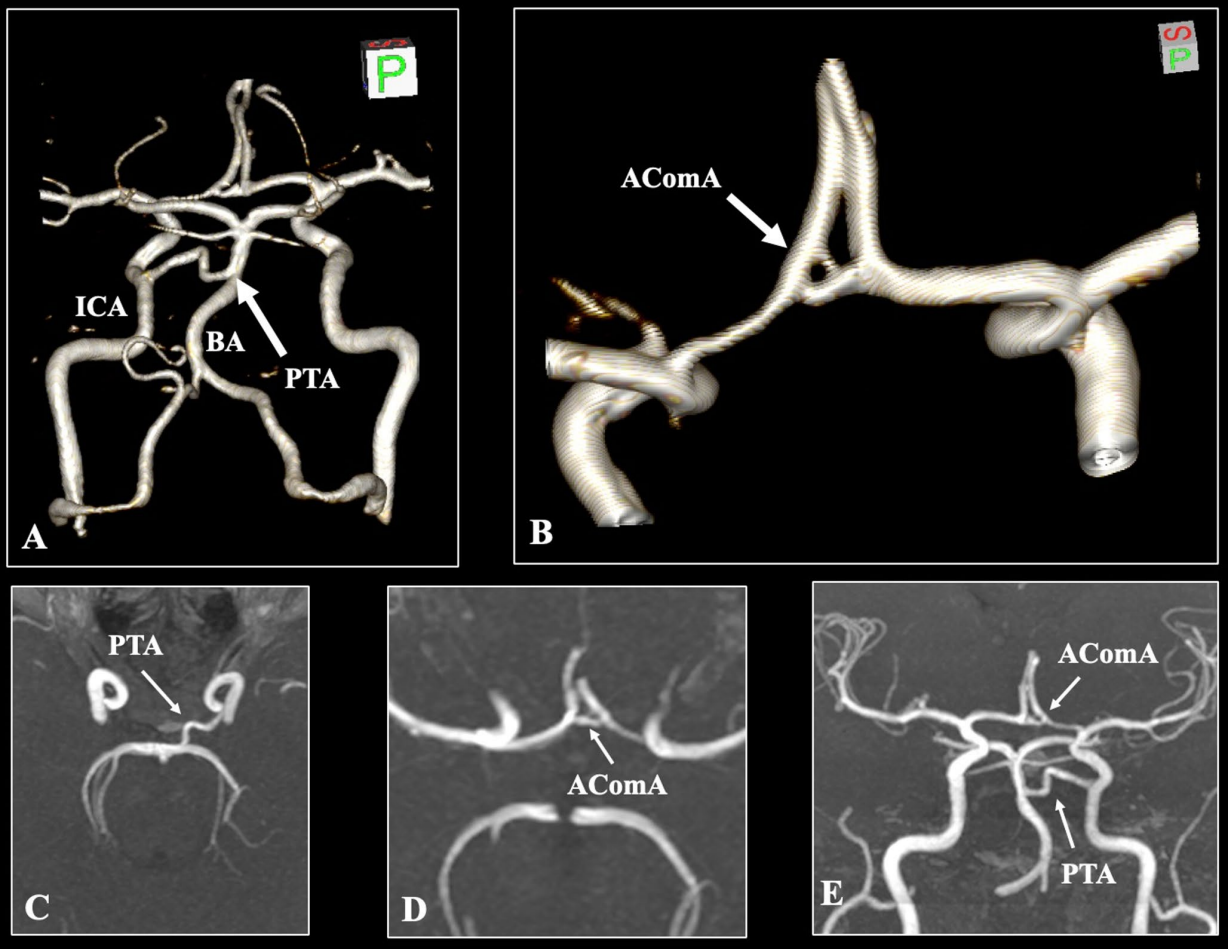
Three-dimensional reconstruction (**A**, **B**), axial slices (**C**, **D**), and coronal slice (**E**) focused on the arterial variants. The persistent trigeminal artery (PTA) is indicated as the anastomosis between the basilar artery (BA) and the internal carotid artery (ICA). This variant is associated with the partial duplication of the anterior communicating artery (AComA)

The bilateral vertebral arteries are conventionally fused in the posterior circulation to form the basilar artery (BA). The BA, exhibiting a diameter of 3.1 mm, followed a standard course; however, upon reaching a length of 18.1 mm, it emitted a variant vessel. This vessel, measuring 1.9 mm in diameter and 19.2 mm in length, anastomosed with the cavernous segment of the left internal carotid artery (ICA), thus classifying it as a PTA. The current case of PTA followed a variable course with curvature in the form of a steep upward peak. The BA tip was situated 8.4 mm distally to the PTA, and the superior cerebellar and posterior cerebral arteries (SCA and PCA) were typically identified (Figs. 1 and 2).

The ICAs typically give rise to the ACAs and MCAs in the anterior circulation. Following a length of 11.4 mm, the bilateral ACAs were anastomosed with the anterior communicating artery (AComA). The vessel exhibited a variant morphology characterized by partial duplication. Two branches of the AComA were observed from the left ACA. The superior branch had a diameter of 1.0 mm and a length of 1.4 mm, whereas the inferior branch had a diameter of 1.5 mm and a length of 2.1 mm. Both branches converged to form a single vessel with a diameter of 1.9 mm and a length of 1.4 mm, subsequently anastomosing with the right ACA (Figs. 1 and 2).

## Discussion

Padget (1948) delineated four types of carotid-vertebrobasilar anastomoses that emerge during the fifth week of gestation, corresponding to an embryonic length of 3–5 mm [5]. The persistent transverse artery (PTA) represents the highest classification of these vessels and is the most frequently encountered variant following birth [8]. The most recent meta-analysis concerning the PTA encompassed 39 studies involving 110,866 patients. The findings indicated that the prevalence of PTA is higher in female patients and is predominantly located on the left side, with the Asian population demonstrating a particularly elevated frequency [2]. The two major types of PTA are the lateral type (similar to the current case) and the medial type, while PTA variants can be recorded as the cerebellar arteries arising from the internal carotid system. Both PTAs and their variants are rare structures. Still, their persistence can significantly affect neurosurgical procedures near the Gasserian ganglion or cavernous sinus and lead to hemorrhagic or ischemic complications if preoperative knowledge is lacking [2]. Furthermore, due to the close relationship with the trigeminal nerve, trigeminal neuralgia has been associated with the presence of a PTA [2]. Treatment with a percutaneous approach to the Gasserian ganglion should occur after an MRA evaluation to rule out the presence of a PTA [2]. Computed tomography angiography could also easily depict the vessel.

The AComA complex exhibits high morphological variability. Embryologically, in an 18-mm embryo, the AComA appears in a plexiform shape. Arterial fenestration or duplication can be created due to the incomplete arteries’ fusion or the persistence of the plexiform primitive shape of the AComA [1]. Triantafyllou et al. [7] identified several variations during their meta-analysis, listed in order of decreasing frequency: hypoplastic AComA (8%), fenestration (5%), absent (4.6%), AComA of different shapes (4.5%), duplication (4.3%), and triplication (0.7%) [7]. The fenestration of the AComA has garnered attention as an interesting variation due to its association with aneurysm formation. However, Uchino et al. [10] pointed out this variant is often misdiagnosed. They presented a case of true tiny fenestration using MRA, emphasizing that true fenestration is a rare entity that should not be confused with partial or complete vessel duplication. Specifically, it can be easily misdiagnosed through computed tomography angiography and its three-dimensional reconstruction [10]. Similarly to the current AComA variant, Endo et al. [3] reported the presence of partial duplication, highlighting that only few cases were described in the current literature [3, 4, 10]. Therefore, the current AComA variant should be categorized as a partial duplication per Uchino et al. [10]. Clinically, the AComA is the most common site for aneurysm formation. This malformation is increased in presence of an anatomical variation [7]. Consequently, the artery is frequently approached by neurosurgeons or neuroradiologists for various treatments. The vessel’s morphological variability should be considered before interventional procedures [7]. In the current case, we recorded the partial duplication with MRA of 1.5 Tesla, while Endo et al. [3, 4] identified the same variant with 1.5 and 3 Tesla MRA. However, according to Uchino et al. [10], computed tomography angiography and its three-dimensional reconstruction may not be able to depict this variant.

In the current literature, carotid-vertebrobasilar anastomoses coexist with other variants of the cerebral arterial circle. In the current case, we described the coexistence of PTA with AComA partial duplication, creating a unique arterial anatomy. Uchino et al. [9] identified the coexistence of PTA along with type 2 proatlantal intersegmental artery during MRA scan of an 83-year-old male patient. Radoi et al. [6] recorded the persistence of a primitive olfactory artery along with an azygos pericallosal artery during computed tomography angiography of a 71-year-old male patient. Recently, Endo et al. [4] observed a primitive olfactory artery coexisting with an accessory middle cerebral artery and a partial duplication of the AComA.

In conclusion, we have reported the coexistence of the PTA alongside the partial duplication of the AComA as diagnosed through MRA. Neurosurgeons and neuroradiologists must preoperatively identify both the typical and rare variants of the cerebral arterial circle. Examining three-dimensional volume rendering images proves beneficial for a comprehensive understanding of the anatomy in this region.

## Data Availability

No datasets were generated or analysed during the current study.
